# Supplementary material for: Mediation of Healthy Behaviour on the Association of Frailty with Respiratory Diseases Mortality among 0.4 Million Participants: A Prospective Cohort Study from UK Biobank
Source: Nutrients. 2022 Nov 27;14(23):5046. doi: 10.3390/nu14235046 (PMC9736014; doi:10.3390/nu14235046)
Supplement: Supplementary file 1 [file nutrients-14-05046-s001.zip › nutrients-2053492-supplementary.pdf]

Supplementary appendix

This appendix formed part of the original submission.

Table S1. The numbers (percentages) of participants with missing covariates 2

Table S2. Scoring system for the frailty phenotype and healthy behaviour indices. 3

Figure S1. Directed acyclic graph (DAG) of the effect of the frailty phenotype (exposure) on respiratory diseases mortality (outcome). 5

Table S3. Sensitivity analyses of associations between frailty and respiratory disease mortality. 6

Table S4. Associations between indexes of frailty and respiratory disease mortality. 7

Figure S2. Associations between frailty and respiratory diseases mortality by healthy behavior. 8

Table S5. Associations of healthy behaviour with respiratory disease mortality. 9

Table S6. Sensitivity analyses of joint association of healthy behaviour and frailty with respiratory disease mortality. 10

Table S7. Associations of frailty with respiratory diseases mortality by gender and age..................... 11

Figure S3. The joint associations of healthy behaviour and frailty respiratory diseases mortality by gender and age. 13

Reference 14

Table S1. The numbers (percentages) of participants with missing covariates

Covariates	n	%
Townsend deprivation index	623	0.12%
Race and ethnicity	2776	0.55%
General health	3486	0.69%
Cancer	2774	0.55%
Diabetes	2615	0.52%
Poor psychological status	2471	0.49%
Cardiovascular disease	2222	0.44%
Family history	9848	1.96%
Sleep duration	4214	0.84%
Tea intake	2192	0.44%
Frailty phenotype	36931	7.35%
Healthy behaviours	52910	10.53%

Table S2. Scoring system for the frailty phenotype and healthy behaviour indices.

Variable	UK Biobank question	UK Biobank data field	Coded
**Frailty phenotype**			
Weight loss [1-4]	"Compared with one year ago, has your weight changed?"	2306	Lost weight = 1 Other = 0
Exhaustion [1-4]	"Over the past two weeks, how often have you felt tired or had little energy?"	120107	More than half the days = 1 Nearly every day = 1 Other = 0
Physical activity [1-4]	"In the last 4 weeks did you spend any time doing the following [activities]?<br>How many times in the last 4 weeks did you do light DIY?	6164<br><br>1011	No physical activity = 1 Light DIY (≤ 1/week) = 1<br>Light DIY (≥ 2-3/week) = 0<br>Heavy DIY = 0<br>Walking for pleasure = 0<br>Other exercises = 0 Strenuous sports = 0
Walking speed [1-4]	"How would you describe your usual walking pace?"	924	Slow = 1<br>Other = 0
Hand-grip strength [1-4]	Measured during the baseline assessment	46 (left hand)<br>47 (right hand)<br>31 (sex)<br>21001 (BMI)	The average of the right and left grip strength stratified by gender and body mass index quartiles defined by Fried et al. [2,3]<br>.
**Healthy behaviours index**			
Body mass index [5,6]	BMI value here is constructed from height and weight measured during the initial Assessment Centre visit	21001	18·5-24·9 kg/m2=1<br>Others=0
Smoking [5,6]	the current/past smoking status of the participant	20116	Never=1,<br>Others=0
Diet score [5,6]	Derived from touchscreen food frequency questionnaire	Vegetables (1289,1299); fruit (1309,1319); fish (1329,1339); type and number of slices/ bowls of bread (1438,1448); cereals (1458,1468); red meat intake (1369,1379, 1389); processed meat (1349)	4-7=1<br>0-3=0

Physical activity [5-7]	International physical activity questionnaire	864, 874, 884, 894, 904, 914	≥735 MET min/week=1 <735 MET min/week=0
Alcohol intake [5,6,8]	Derived from daily and weekly alcohol questionnaire	Red wine (1568, 4407); champagne/ white wine (1578, 4418); beer/ cider (1588, 4429); spirits (1598, 4440), fortified wine intake (1608, 4451); and other alcoholic drinks (5364, 4462)	Female: <5 or >15 g/day=0 Male: <5 or >30 g/day=0 Female: 5-15 g/day=1 Male: 5-30 g/day=1

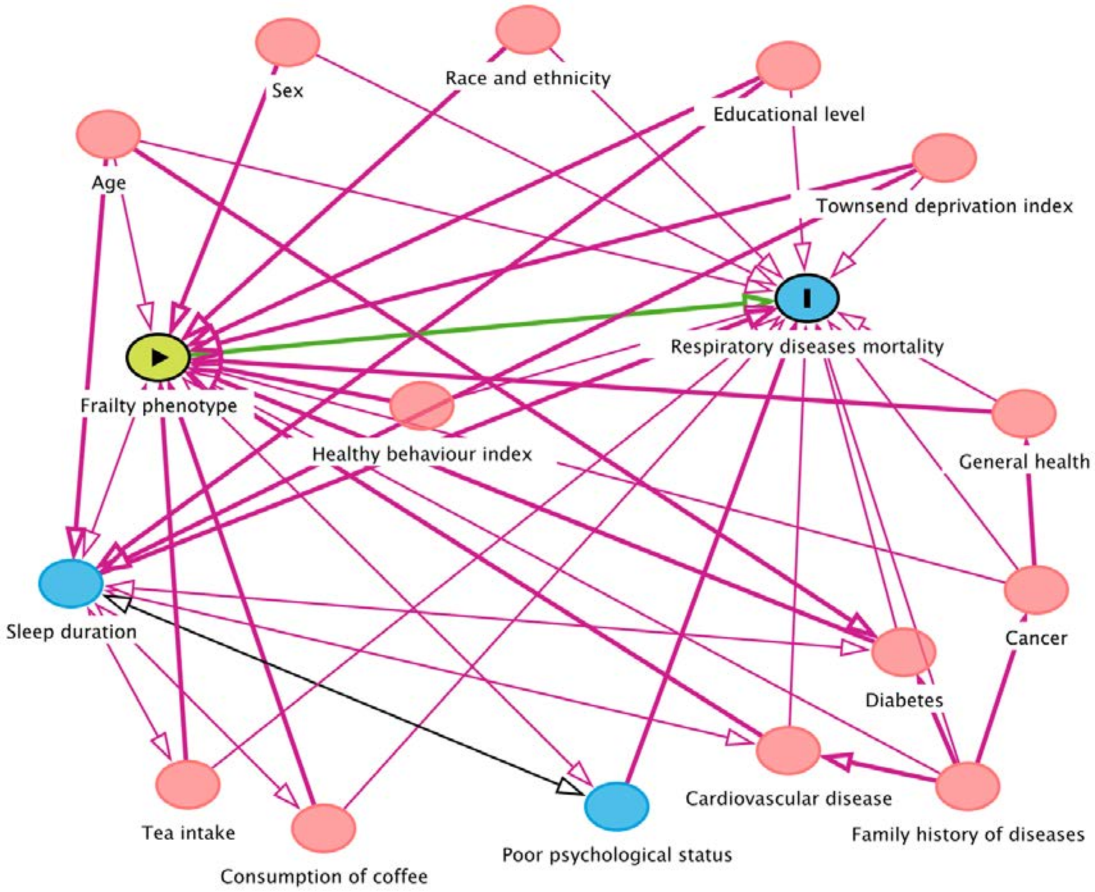

Figure S1. Directed acyclic graph (DAG) of the effect of the frailty phenotype (exposure) on respiratory diseases mortality (outcome).

Ancestors of exposure and outcome, ancestors of outcome, exposure, and outcome.

Table S3. Sensitivity analyses of associations between frailty and respiratory disease mortality.

	Hazard ratio (95% CI)	
	Unadjusted for healthy behaviours	Adjusted for healthy behaviours
**After excluded participants who had an outcome event during the first 5 years of follow-up**		
**Total respiratory diseases**		
Robust	1 (Reference)	1 (Reference)
Pre-frail	1.42 (1.24, 1.62)	1.39 (1.22, 1.59)
Frail	2.65 (2.21, 3.19)	2.52 (2.10, 3.04)
**Influenza and pneumonia**		
Robust	1 (Reference)	1 (Reference)
Pre-frail	1.61 (1.23, 2.12)	1.60 (1.22, 2.11)
Frail	3.13 (2.15, 4.57)	3.05 (2.09, 4.46)
**Chronic lower respiratory diseases**		
Robust	1 (Reference)	1 (Reference)
Pre-frail	1.52 (1.22, 1.91)	1.48 (1.18, 1.86)
Frail	3.28 (2.49, 4.33)	3.07 (2.32, 4.06)
**Multiple imputation by chained equations to impute all missing independent variables**		
**Total respiratory diseases**		
Robust	1 (Reference)	1 (Reference)
Pre-frail	1.44 (1.28, 1.62)	1.41 (1.24, 1.60)
Frail	2.77 (2.38, 3.22)	2.68 (2.26, 3.16)
**Influenza and pneumonia**		
Robust	1 (Reference)	
Pre-frail	1.60 (1.26, 2.02)	1.64 (1.27, 2.12)
Frail	3.13 (2.28, 4.30)	3.27 (2.30, 4.64)
**Chronic lower respiratory diseases**		
Robust	1 (Reference)	
Pre-frail	1.64 (1.35, 2.00)	1.52 (1.23, 1.88)
Frail	3.58 (2.84, 4.52)	3.31 (2.56, 4.26)

Notes: Models all adjusted for age, sex, TDI, educational level, race and ethnicity, general health, cancer, diabetes, cardiovascular disease, poor psychological status, family history, sleep duration, coffee intake, and consumption of tea. HR= hazard ratio. 95% CI=95% confidence interval.

Table S4. Associations between indexes of frailty and respiratory disease mortality.

	Hazard ratio (95% CI)	
	**Unadjusted for healthy behaviours**	**Adjusted for healthy behaviours**
**Total respiratory diseases**		
Low physical activity	1.12 (1.01 ,1.24)	1.09 (0.99 ,1.21)
Low grip strength	1.35 (1.21 ,1.50)	1.34 (1.21 ,1.50)
Slow walking pace	2.52 (2.23 ,2.86)	2.41 (2.12 ,2.74)
Weight loss	1.37 (1.22 ,1.55)	1.39 (1.23 ,1.57)
Exhaustion	0.92 (0.80 ,1.05)	0.92 (0.80 ,1.05)
**Influenza and pneumonia**		
Low physical activity	1.18 (0.96 ,1.45)	1.16 (0.94 ,1.42)
Low grip strength	1.74 (1.40 ,2.17)	1.74 (1.40 ,2.16)
Slow walking pace	2.06 (1.58 ,2.69)	1.99 (1.52 ,2.60)
Weight loss	1.41 (1.10 ,1.79)	1.42 (1.12 ,1.81)
Exhaustion	0.77 (0.58 ,1.04)	0.78 (0.58 ,1.04)
**Chronic lower respiratory diseases**		
Low physical activity	1.21 (1.04 ,1.40)	1.17 (1.01 ,1.36)
Low grip strength	1.14 (0.97 ,1.33)	1.13 (0.96 ,1.33)
Slow walking pace	3.63 (3.03 ,4.35)	3.42 (2.85 ,4.10)
Weight loss	1.56 (1.31 ,1.85)	1.59 (1.33 ,1.89)
Exhaustion	0.97 (0.81 ,1.16)	0.97 (0.81 ,1.16)

Notes: Models all adjusted for age, sex, TDI, educational level, race and ethnicity, general health, cancer, diabetes, cardiovascular disease, poor psychological status, family history, sleep duration, coffee intake, and consumption of tea. HR= hazard ratio. 95% CI=95% confidence interval.

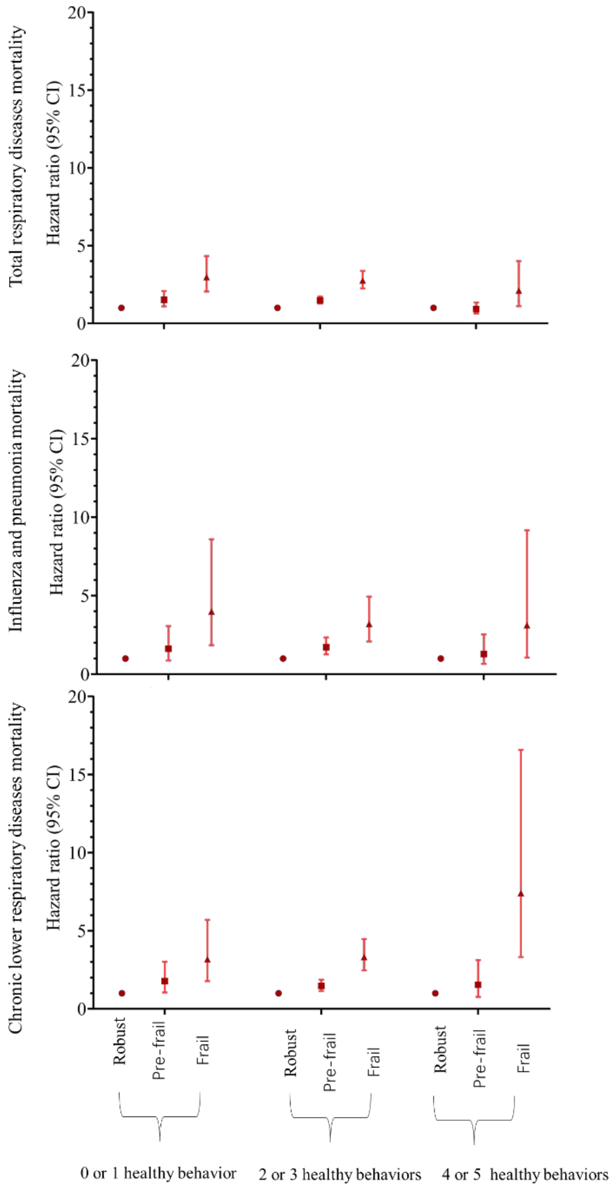

Figure S2. Associations between frailty and respiratory diseases mortality by healthy behavior.

Models all adjusted for age, sex, TDI, educational level, race and ethnicity, general health, cancer, diabetes, cardiovascular disease, poor psychological status, family history, sleep duration, coffee intake, and consumption of tea. HR= hazard ratio. 95% CI=95% confidence interval.

Table S5. Associations of healthy behaviour with respiratory disease mortality.

	Hazard ratio (95% CI)
**Total respiratory diseases**	
0 or 1 healthy behaviour	1 (Reference)
2 or 3 healthy behaviours	0.75 (0.67, 0.85)
4 or 5 healthy behaviours	0.46 (0.37, 0.57)
**Influenza and pneumonia**	
0 or 1 healthy behaviour	1 (Reference)
2 or 3 healthy behaviours	0.73 (0.57, 0.95)
4 or 5 healthy behaviours	0.64 (0.44, 0.95)
**Chronic lower respiratory diseases**	
0 or 1 healthy behaviour	1 (Reference)
2 or 3 healthy behaviours	0.74 (0.63, 0.89)
4 or 5 healthy behaviours	0.28 (0.19, 0.41)

Models all adjusted for age, sex, TDI, educational level, race and ethnicity, general health, cancer, diabetes, cardiovascular disease, poor psychological status, family history, sleep duration, coffee intake, and consumption of tea. HR= hazard ratio. 95% CI=95% confidence interval.

Table S6. Sensitivity analyses of joint association of healthy behaviour and frailty with respiratory disease mortality.

	Hazard ratio (95% CI)	
	**Sensitivity analyses [1]**	**Sensitivity analyses [2]**
**Total respiratory diseases**		
Robust & 4 or 5 healthy behaviours	1 (reference)	1 (reference)
Robust & 2 or 3 healthy behaviours	1.25 (0.91, 1.72)	1.26 (0.93, 1.70)
Robust & 0 or 1 healthy behaviour	1.71 (1.13, 2.58)	1.68 (1.14, 2.49)
Pre-frail & 4 or 5 healthy behaviours	0.93 (0.63, 1.38)	0.95 (0.66, 1.37)
Pre-frail & 2 or 3 healthy behaviours	1.85 (1.36, 2.51)	1.86 (1.40, 2.48)
Pre-frail & 0 or 1 healthy behaviour	2.37 (1.69, 3.32)	2.49 (1.82, 3.41)
Frail & 4 or 5 healthy behaviours	2.78 (1.56, 4.94)	2.33 (1.32, 4.10)
Frail & 2 or 3 healthy behaviours	3.25 (2.32, 4.56)	3.50 (2.56, 4.79)
Frail1 & 0 or 1 healthy behaviour	4.18 (2.88, 6.07)	4.59 (3.27, 6.45)
**Influenza and pneumonia**		
Robust & 4 or 5 healthy behaviours	1 (reference)	1 (reference)
Robust & 2 or 3 healthy behaviours	0.99 (0.54, 1.83)	1.01 (0.56, 1.83)
Robust & 0 or 1 healthy behaviour	1.30 (0.57, 2.97)	1.57 (0.74, 3.36)
Pre-frail & 4 or 5 healthy behaviours	1.40 (0.71, 2.76)	1.36 (0.70, 2.64)
Pre-frail & 2 or 3 healthy behaviours	1.63 (0.92, 2.91)	1.76 (1.01, 3.06)
Pre-frail & 0 or 1 healthy behaviour	2.09 (1.09, 3.99)	2.33 (1.26, 4.31)
Frail & 4 or 5 healthy behaviours	4.35 (1.63, 11.64)	4.07 (1.54, 10.75)
Frail & 2 or 3 healthy behaviours	3.00 (1.56, 5.77)	3.39 (1.82, 6.31)
Frail1 & 0 or 1 healthy behaviour	3.71 (1.77, 7.76)	4.55 (2.30, 9.03)
**Chronic lower respiratory diseases**		
Robust & 4 or 5 healthy behaviours	1 (reference)	1 (reference)
Robust & 2 or 3 healthy behaviours	2.76 (1.27, 5.98)	3.12 (1.45, 6.74)
Robust & 0 or 1 healthy behaviour	3.21 (1.31, 7.90)	3.59 (1.48, 8.68)
Pre-frail & 4 or 5 healthy behaviours	1.18 (0.46, 3.00)	1.55 (0.64, 3.78)
Pre-frail & 2 or 3 healthy behaviours	4.01 (1.88, 8.53)	4.52 (2.13, 9.60)
Pre-frail & 0 or 1 healthy behaviour	5.56 (2.54, 12.19)	6.67 (3.07, 14.49)
Frail & 4 or 5 healthy behaviours	6.15 (2.21, 17.11)	5.72 (2.06, 15.90)
Frail & 2 or 3 healthy behaviours	8.28 (3.80, 18.03)	10.09 (4.67, 21.82)
Frail1 & 0 or 1 healthy behaviour	10.15 (4.54, 22.71)	12.70 (5.76, 27.97)

Models all adjusted for age, sex, TDI, educational level, race and ethnicity, general health, cancer, diabetes, cardiovascular disease, poor psychological status, family history, sleep duration, coffee intake, and consumption of tea. [1]Sensitivity analyses of joint association of healthy behaviours and frailty with respiratory disease mortality after excluded participants who had an outcome event during the first 5 years of follow-up; [2]Sensitivity analyses of joint association of healthy behaviours and frailty with respiratory disease mortality after using multiple imputation by chained equations to impute all missing independent variables. HR= hazard ratio. 95% CI=95% confidence interval.

Table S7. Associations of frailty with respiratory diseases mortality by gender and age.

	Hazard ratio (95% CI)	
	**Unadjusted for healthy behaviour index**	**Adjusted for healthy behaviour index**
**Total respiratory diseases**		
**Male**		
Robust	1 (Reference)	1 (Reference)
Pre-frail	1.33 (1.06, 1.67)	1.30 (1.04, 1.62)
Frail	2.85 (2.16, 3.77)	2.66 (2.02, 3.52)
**Female**		
Robust	1 (Reference)	1 (Reference)
Pre-frail	1.49 (1.28, 1.73)	1.47 (1.26, 1.71)
Frail	2.75 (2.23, 3.40)	2.64 (2.14, 3.26)
**<65 years**		
Robust	1 (Reference)	1 (Reference)
Pre-frail	1.48 (1.24, 1.78)	1.45 (1.21, 1.74)
Frail	3.18 (2.51, 4.02)	2.99 (2.37, 3.79)
**≥65 years**		
Robust	1 (Reference)	1 (Reference)
Pre-frail	1.41 (1.19, 1.68)	1.39 (1.17, 1.66)
Frail	2.47 (1.94, 3.14)	2.38 (1.87, 3.03)
**Influenza and pneumonia**		
**Male**		
Robust	1 (Reference)	1 (Reference)
Pre-frail	1.97 (1.21, 3.20)	1.95 (1.20, 3.18)
Frail	3.54 (1.92, 6.52)	3.45 (1.86, 6.38)
**Female**		
Robust	1 (Reference)	1 (Reference)
Pre-frail	1.54 (1.14, 2.09)	1.52 (1.12, 2.06)
Frail	3.39 (2.20, 5.22)	3.24 (2.10, 5.01)
**<65 years**		
Robust	1 (Reference)	1 (Reference)
Pre-frail	1.79 (1.25, 2.56)	1.75 (1.22, 2.51)
Frail	4.26 (2.65, 6.86)	3.98 (2.46, 6.43)
**≥65 years**		
Robust	1 (Reference)	1 (Reference)
Pre-frail	1.54 (1.07, 2.22)	1.55 (1.08, 2.24)
Frail	2.57 (1.53, 4.32)	2.64 (1.56, 4.44)
**Chronic lower respiratory diseases**		
**Male**		
Robust	1 (Reference)	1 (Reference)
Pre-frail	1.24 (0.87, 1.76)	1.19 (0.84, 1.69)
Frail	3.29 (2.21, 4.92)	2.94 (1.96, 4.39)
**Female**		
Robust	1 (Reference)	1 (Reference)
Pre-frail	1.76 (1.35, 2.30)	1.73 (1.33, 2.26)
Frail	3.64 (2.62, 5.05)	3.51 (2.53, 4.88)
**<65 years**		
Robust	1 (Reference)	1 (Reference)

Pre-frail	1.32 (1.00, 1.75)	1.30 (0.98, 1.72)
Frail	2.79 (1.98, 3.93)	2.62 (1.86, 3.70)
**≥65 years**		
Robust	1 (Reference)	1 (Reference)
Pre-frail	1.93 (1.39, 2.67)	1.87 (1.35, 2.59)
Frail	4.63 (3.16, 6.78)	4.28 (2.91, 6.28)

Models all adjusted for age, sex, TDI, educational level, race and ethnicity, general health, cancer, diabetes, cardiovascular disease, poor psychological status, family history, sleep duration, coffee intake, and consumption of tea. HR= hazard ratio. 95% CI=95% confidence interval.

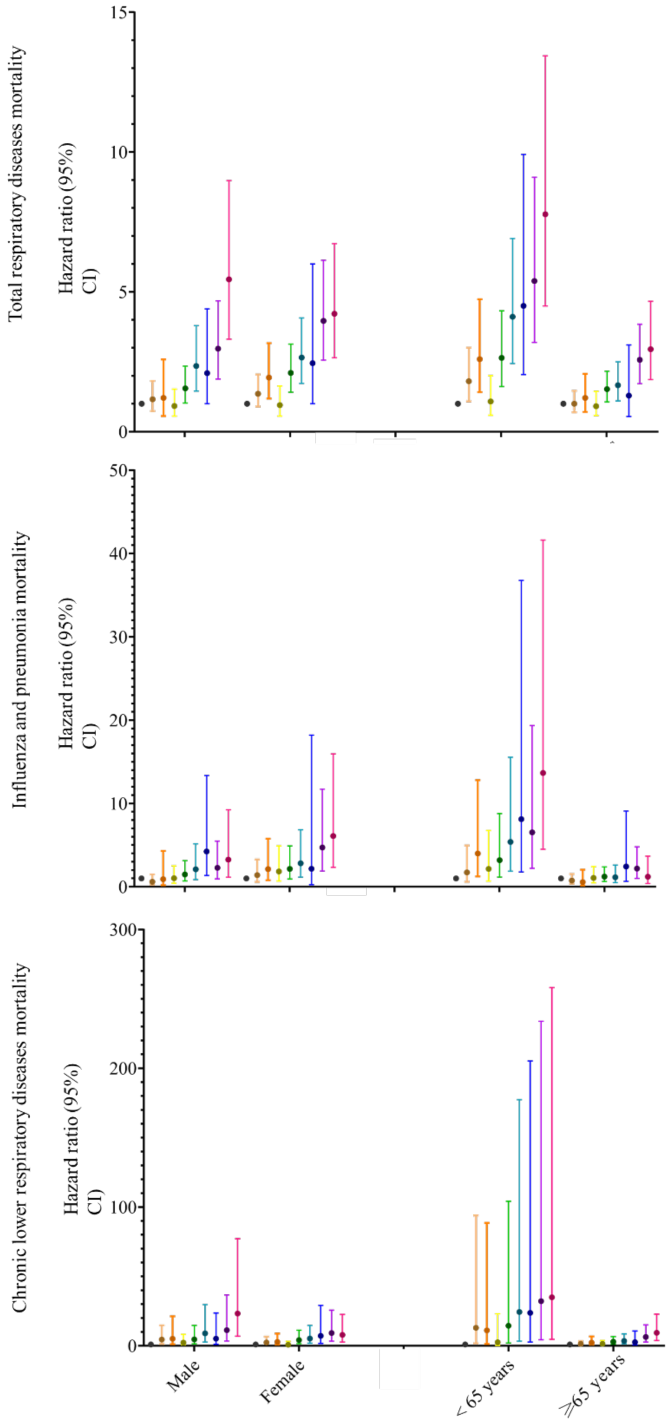

Figure S3. The joint associations of healthy behaviour and frailty respiratory diseases mortality by gender and age.

Models all adjusted for age, sex, TDI, educational level, race and ethnicity, general health, cancer, diabetes, cardiovascular disease, poor psychological status, family history, sleep duration, coffee intake, and consumption of tea. Nine joint groups were presented in the following order: Robust & 4 or 5 healthy behaviours (reference group), Robust & 2 or 3 healthy behaviours, Robust & 0 or 1 healthy behaviour, Pre-frail & 4 or 5 healthy behaviours, Pre-frail & 2 or 3 healthy behaviours, Pre-frail & 0 or 1 healthy behaviour, Frail & 4 or 5 healthy behaviours, Frail & 2 or 3 healthy behaviours Frail1 & 0 or 1 healthy behaviour in four subgroups. HR= hazard ratio. 95% CI=95% confidence interval.

Reference

1. Hanlon, P.; Nicholl, B.I.; Jani, B.D.; Lee, D.; McQueenie, R.; Mair, F.S. Frailty and pre-frailty in middle-aged and older adults and its association with multimorbidity and mortality: a prospective analysis of 493 737 UK Biobank participants. *The Lancet. Public health* **2018**, *3*, e323-e332, doi:10.1016/s2468-2667(18)30091-4.
2. Fried, L.P.; Tangen, C.M.; Walston, J.; Newman, A.B.; Hirsch, C.; Gottdiener, J.; Seeman, T.; Tracy, R.; Kop, W.J.; Burke, G., et al. Frailty in older adults: evidence for a phenotype. *The journals of gerontology. Series A, Biological sciences and medical sciences* **2001**, *56*, M146-156, doi:10.1093/gerona/56.3.m146.
3. Petermann-Rocha, F.; Hanlon, P.; Gray, S.R.; Welsh, P.; Gill, J.M.R.; Foster, H.; Katikireddi, S.V.; Lyall, D.; Mackay, D.F.; O'Donnell, C.A., et al. Comparison of two different frailty measurements and risk of hospitalisation or death from COVID-19: findings from UK Biobank. *BMC medicine* **2020**, *18*, 355, doi:10.1186/s12916-020-01822-4.
4. Mutz, J.; Choudhury, U.; Zhao, J.; Dregan, A. Frailty in individuals with depression, bipolar disorder and anxiety disorders: longitudinal analyses of all-cause mortality. *BMC medicine* **2022**, *20*, 274, doi:10.1186/s12916-022-02474-2.
5. Bountziouka, V.; Musicha, C.; Allara, E.; Kaptoge, S.; Wang, Q.; Angelantonio, E.D.; Butterworth, A.S.; Thompson, J.R.; Danesh, J.N.; Wood, A.M., et al. Modifiable traits, healthy behaviours, and leukocyte telomere length: a population-based study in UK Biobank. *The Lancet. Healthy longevity* **2022**, *3*, e321-e331, doi:10.1016/s2666-7568(22)00072-1.
6. Yang, G.; Cao, X.; Li, X.; Zhang, J.; Ma, C.; Zhang, N.; Lu, Q.; Crimmins, E.M.; Gill, T.M.; Chen, X., et al. Association of Unhealthy Lifestyle and Childhood Adversity With Acceleration of Aging Among UK Biobank Participants. *JAMA network open* **2022**, *5*, e2230690, doi:10.1001/jamanetworkopen.2022.30690.
7. Fan Mengyu, Lyu Jun, He Pingping. Chinese guidelines for data processing and analysis concerning the International Physical Activity Questionnaire. Chinese Journal of Epidemiology 2014; 35(08): 961-964.
8. Zhang, Y.B.; Chen, C.; Pan, X.F.; Guo, J.; Li, Y.; Franco, O.H.; Liu, G.; Pan, A. Associations of healthy lifestyle and socioeconomic status with mortality and incident cardiovascular disease: two prospective cohort studies. *BMJ (Clinical research ed.)* **2021**, *373*, n604, doi:10.1136/bmj.n604.
